# Concurrent Breast Cancer and Vulvar Leiomyoma in a 62-Year-Old Patient: A Comprehensive Case Report

**DOI:** 10.7759/cureus.83420

**Published:** 2025-05-03

**Authors:** Cristina Bacalam, Daniel Sur, Ovidiu Bochis, Rares Buiga

**Affiliations:** 1 Department of Medical Oncology, The Oncology Institute "Prof. Dr. Ion Chiricuţă" Cluj-Napoca, Cluj-Napoca, ROU; 2 Department of Medical Oncology, Iuliu Hațieganu University of Medicine and Pharmacy, Cluj-Napoca, ROU; 3 Department of Pathology, The Oncology Institute "Prof. Dr. Ion Chiricuţă" Cluj-Napoca, Cluj-Napoca, ROU

**Keywords:** breast cancer, case report, differential diagnosis, vulvar leiomyoma, vulvar mass

## Abstract

The detection of incidental masses in cancer patients, such as vulvar leiomyomas, is rare and can complicate the diagnostic process. We report a case of a 62-year-old woman who presented with a large, neglected breast tumor, which raised clinical suspicion of metastatic breast cancer. A computed tomography scan showed an osteosclerotic pelvic lesion, raising concern for bone metastasis. A whole-body positron emission tomography scan revealed no bone metastasis but detected a hypermetabolic left vulvar mass, presumed to be a Bartholin cyst. Surgical excision confirmed it to be a vulvar leiomyoma. The patient underwent neoadjuvant chemotherapy for breast cancer, followed by a left radical mastectomy with significant residual tumor burden. Currently, the patient is receiving radiotherapy along with adjuvant hormonal treatment, and CDK4/6 inhibitor therapy is scheduled after the radiotherapy. When found incidentally during cancer staging, vulvar leiomyomas can cause diagnostic confusion. This case highlights the importance of a comprehensive and multidisciplinary differential diagnostic approach in the oncological assessment of cancer patients to avoid misdiagnosis and guide appropriate treatment strategies.

## Introduction

Vulvar leiomyoma is a rare, benign, smooth muscle tumor of the female genital tract. It accounts for 0.03% of all gynecological tumors and 0.07% of vulvar neoplasms [1]. Fewer than several hundred cases have been documented in the medical literature. Vulvar leiomyomas typically occur in women of reproductive age (most commonly in the 30s-40s) and usually involve the labia majora or clitoral area [2,3]. The pathogenesis is not fully understood, but hormonal influences are suspected. These tumors rarely appear before menarche and often regress after menopause, suggesting estrogen and progesterone may promote their growth [2]. Although vulvar leiomyomas are generally considered benign with a low risk of recurrence, rare cases of recurrent leiomyomas have been documented [4]. Specifically, some have been initially misdiagnosed as other soft tissue tumors, resulting in incomplete excision and subsequent recurrence. A recent study detailed a case of recurrent vulvar leiomyoma that was misidentified as a peripheral nerve sheath tumor, highlighting the crucial role of immunohistochemistry in achieving an accurate diagnosis. Wide local excision with clear margins is recommended to minimize the risk of recurrence, especially in cases where prior incomplete removal may have led to regrowth [4]. This report describes the unexpected discovery of a vulvar leiomyoma in a postmenopausal patient undergoing staging for breast cancer.

## Case presentation

**Figure 1:**
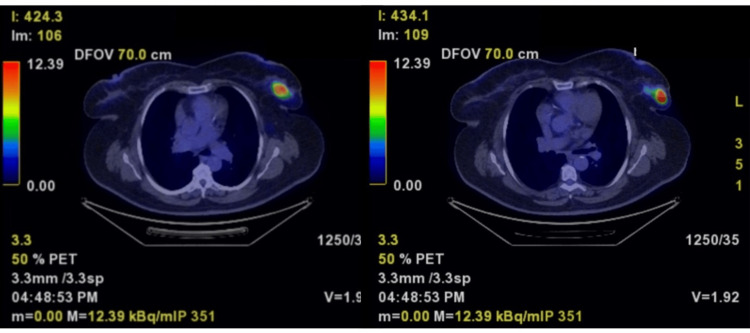
PET scan showing two adjacent hypermetabolic breast tumors (standardized uptake value = 8) measuring 35 × 20 mm and 30 × 15 mm, located in the upper outer quadrant and inferior outer quadrant, respectively, of the left mammary gland.

**Figure 2:**
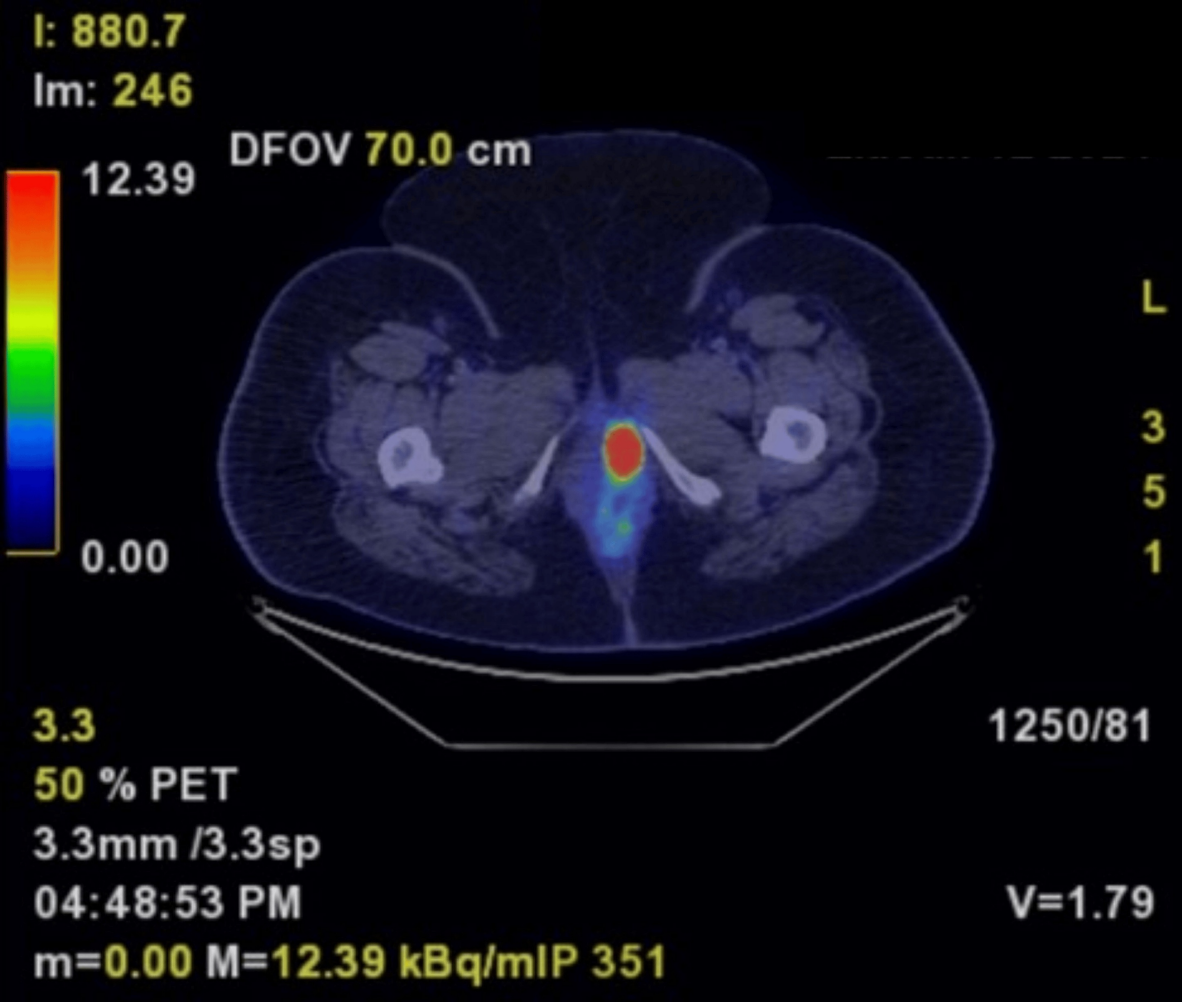
The PET scan showed an active mass in the left vulvar region, measuring 38 × 25 mm, with intense fluorodeoxyglucose uptake.

A 62-year-old woman presented to our clinic with a large, neglected breast mass, raising clinical suspicion for advanced breast cancer. A staging computed tomography scan revealed an osteosclerotic pelvic bone lesion, raising concern for bone metastasis. A whole-body positron emission tomography (PET) scan was used to assess the extent of the disease. The PET scan revealed no bone metastasis but did detect a hypermetabolic left vulvar mass, measuring 38 × 25 mm (Figures 1, 2). Notably, the computed tomography scan detected no vulvar abnormalities, and the asymptomatic lump was only discovered after several gynecologic examinations. The mass was presumed to be a Bartholin cyst. A surgical excision was performed.

**Figure 3:**
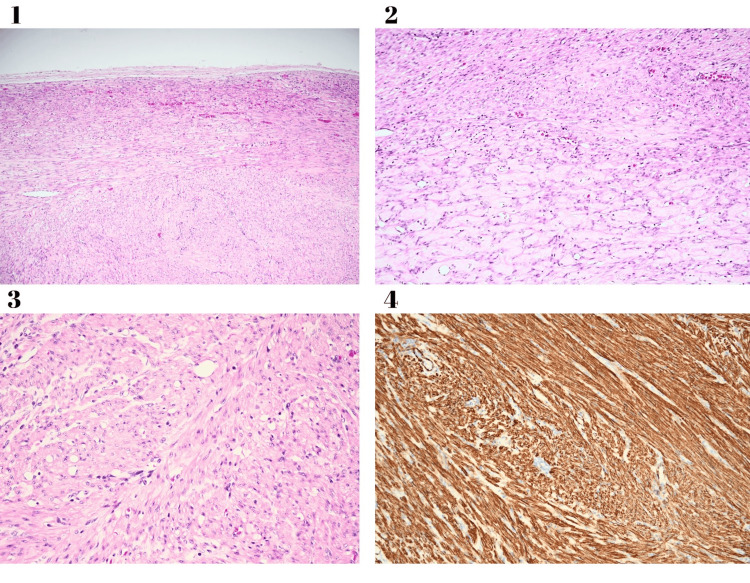
Histological findings of the benign tissue specimen. Leiomyoma (hematoxylin-eosin stain, 100x) with smooth noninvasive borders (1). Leiomyoma (hematoxylin-eosin stain, 200x) with focal hyalinization (2). Leiomyoma (hematoxylin-eosin stain, 400x) with smooth muscle cells without significant atypia, grouped in wide bundles that intersect at right angles (3). Leiomyoma (h-Caldesmon, 200x) with diffuse and intense positivity (4).

**Figure 4:**
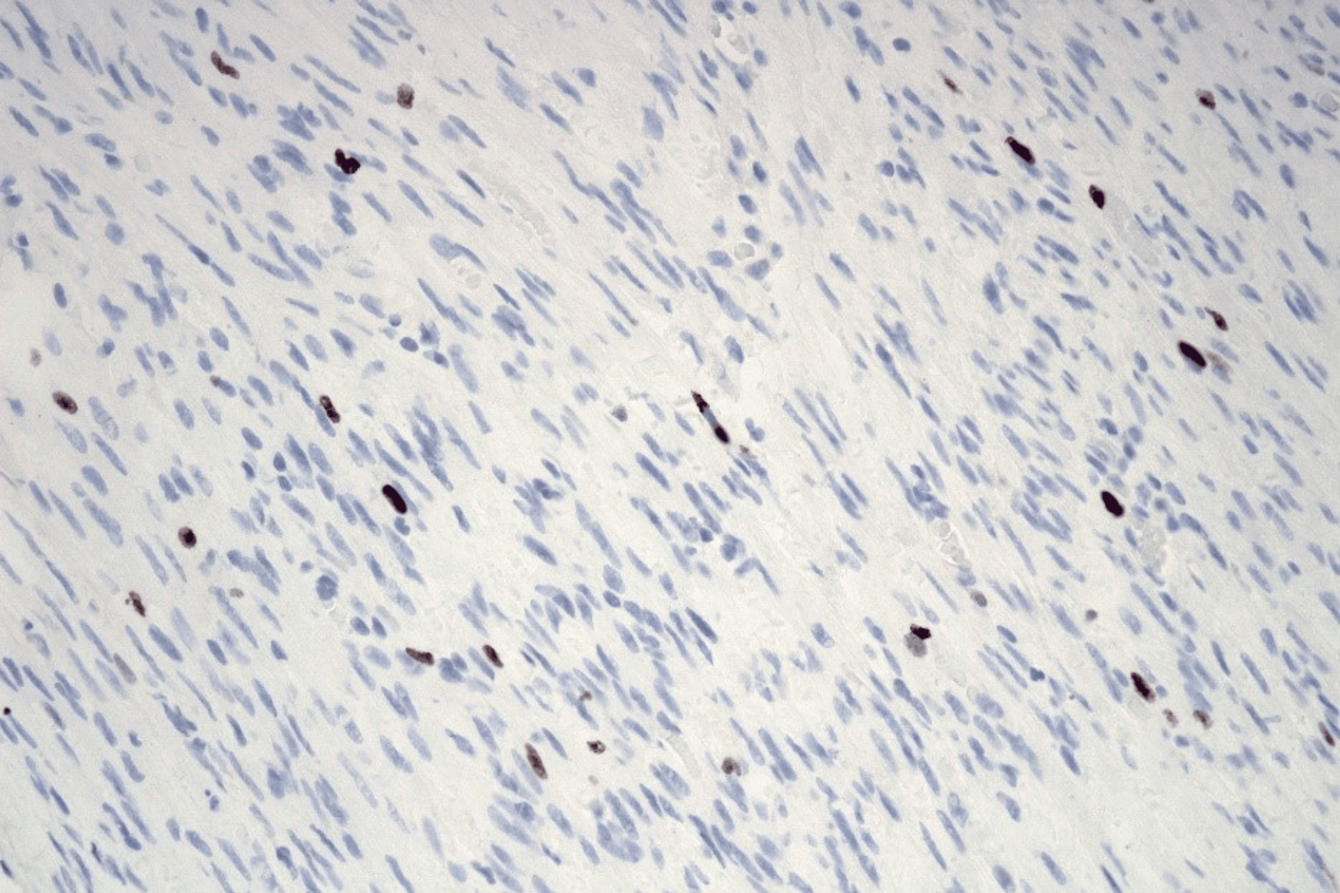
Leiomyoma (Ki67, 400x). Although the global Ki67 proliferation index does not exceed 2%, the tumor shows some proliferative foci, such as the one in this image, in which the Ki67 index can exceed 10%.

Histopathological examination revealed a fleshy, solid cut surface with no cystic formations observed. The microscopic analysis identified a benign tumor characterized by smooth muscle cells without significant atypia, grouped in wide bundles that intersect at right angles, smooth noninvasive borders, and focal hyalinization (Figure 3). Although the global Ki67 proliferation index does not exceed 2%, the tumor shows some proliferative foci, such as the one in this image, in which the Ki67 index can exceed 10% (Figure 4). The final diagnosis was a benign vulvar leiomyoma.

The patient underwent sequential neoadjuvant chemotherapy that included four cycles of epirubicin and cyclophosphamide, followed by four cycles of docetaxel, experiencing no significant toxicities. Subsequently, she had a left radical mastectomy. Histopathological examination showed a notable residual tumor burden after the neoadjuvant chemotherapy, with final staging classified as ypT2 N2a. Currently, the patient is receiving radiotherapy and adjuvant hormonal treatment, with CDK4/6 inhibitor therapy planned to start after the radiotherapy is completed. During this time, no evidence of vulvar leiomyoma recurrence was observed.

## Discussion

This case contributes to the growing body of evidence demonstrating the potential for benign smooth muscle tumors to exhibit hypermetabolic activity on PET scans, often mimicking malignancies. In a prior study by Chura et al. [5], PET-positive leiomyomas were misinterpreted as malignant lesions, leading to unnecessary or extensive surgical interventions. Although leiomyomas typically exhibit mild to moderate fluorodeoxyglucose (FDG) uptake, increased glucose metabolism may be attributed to factors such as enhanced vascular proliferation, overexpression of glucose transporters, or increased perfusion due to inflammatory responses [5,6].

Vulvar leiomyomas, though rare, have been well-documented as part of the spectrum of extrauterine smooth muscle tumors [1,3]. Unlike their uterine counterparts, vulvar leiomyomas often present as well-circumscribed, slow-growing masses that can be mistaken for Bartholin's cysts. Their infrequency leads to delays in diagnosis; in this case, the lesion was only discovered after repeated clinical examinations. Imaging techniques like ultrasound and MRI are most helpful for characterization. MRI is particularly useful in distinguishing benign leiomyomas from leiomyosarcomas based on signal intensity and the absence of necrosis or significant mitotic activity [7].

A notable aspect of this case is the coexistence of vulvar leiomyoma with an advanced breast malignancy. While breast cancer and leiomyomas are not typically associated, some studies suggest a potential hormonal link. Leiomyomas express estrogen and progesterone receptors, and systemic hormonal changes may influence their growth [1,8]. The possible impact of breast cancer treatment, particularly tamoxifen therapy, on smooth muscle tumors has also been explored, with reports suggesting that tamoxifen may promote the growth of uterine and possibly extrauterine leiomyomas due to its estrogen-agonist activity in non-breast tissues [9,10]. However, this patient had not received prior hormonal therapy.

The differential diagnosis of vulvar leiomyoma in the context of a known malignancy is particularly significant. Given the high metabolic activity observed on PET scans in this case, an initial concern for metastatic disease was reasonable. However, as illustrated in prior reports, PET positivity alone is insufficient to determine malignancy, and histological confirmation remains essential [5].

The presence of proliferative foci in our case, where the Ki-67 index exceeded 10% in certain areas, may suggest a subset of leiomyomas with increased growth potential that requires further investigation in clinical practice. These uterine smooth muscle tumors generally show very low Ki-67 proliferation activity. In leiomyomas, the Ki-67 index typically ranges from 0% to 5%, with most cases showing very low proliferative activity. In contrast, leiomyosarcomas often exhibit Ki-67 positivity >10%, with some cases reaching 20-30%, correlating with aggressive growth and poorer prognosis. Studies have shown that higher Ki-67 expression in leiomyomas may be associated with increased bleeding, anemia, and pain severity, though it remains significantly lower than in malignant counterparts [11,12].

Although rare, metastases from breast cancer to the vulva have been documented in the literature [13,14]. Invasive lobular carcinoma, in particular, has a known propensity for unusual metastatic sites, including the gynecologic tract [15]. Reports describe cases where vulvar metastases presented as discrete nodules or diffuse thickening, often mimicking primary vulvar malignancies or benign conditions such as Bartholin cysts [16,17]. Clinical symptoms can include vulvar swelling, ulceration, pain, or even abnormal vaginal discharge. Imaging modalities such as MRI and PET/CT can assist in identifying suspicious lesions [17]. Still, a definitive diagnosis requires histopathological confirmation, using immunohistochemical markers specific to breast carcinoma. A key distinguishing factor between metastatic breast carcinoma and primary vulvar tumors (including leiomyomas) is the pattern of cellular invasion. Metastatic breast cancer often exhibits infiltrative growth, cytologic atypia, and frequent mitotic activity, which are absent in benign leiomyomas. Furthermore, while vulvar leiomyomas show smooth muscle differentiation on immunohistochemistry, breast cancer metastases retain epithelial markers characteristic of their primary origin [14].

Vulvar leiomyomas are benign with low recurrence risk, though rare cases of recurrence exist [4]. The mechanism underlying recurrence in vulvar leiomyomas is not entirely understood. While uterine leiomyomas tend to regress after menopause due to hormonal withdrawal, some reports suggest that extrauterine leiomyomas may persist or even recur in postmenopausal women, indicating a potential role for alternative growth mechanisms beyond hormonal influence [4,8]. Given these findings, long-term follow-up is advisable for patients with a history of vulvar leiomyomas, particularly those with larger tumors or incomplete prior excisions.

## Conclusions

This case underscores the importance of a thorough diagnostic workup in oncologic patients presenting with unexpected hypermetabolic lesions. It also highlights the need for increased awareness of rare benign tumors, such as vulvar leiomyomas, which can mimic more serious conditions on advanced imaging. Documenting such cases in oncology literature will continue to inform future best practices, ensuring optimal care for patients who navigate between two seemingly unrelated diagnoses. Given the excellent prognosis associated with surgical excision of vulvar leiomyomas, early recognition and accurate diagnosis are crucial to prevent unnecessary radical treatments and optimize patient outcomes.
